# Commercial perspectives: Genome editing as a breeding tool for health and well-being in dairy cattle*

**DOI:** 10.3168/jdsc.2023-0481

**Published:** 2024-09-02

**Authors:** Tad S. Sonstegard, Julio M. Flórez, José Fernando Garcia

**Affiliations:** 1 Acceligen Inc., Eagan, MN 55121; 2 Department of Preventive Veterinary Medicine and Animal Reproduction, School of Agricultural and Veterinary Sciences, São Paulo State University (UNESP), Jaboticabal, 14884-900, Brazil; 3 Department of Animal Production and Health, School of Veterinary Medicine, São Paulo State University (UNESP), Araçatuba, 16050-680, Brazil; 4 International Atomic Energy Agency Collaborating Centre on Animal Genomics and Bioinformatics, Araçatuba, 16050-680, Brazil

## Abstract

Genome editing is the latest breeding tool capable of accelerating the rate of genetic improvement for health and well-being traits in food animals. It enables the introduction of beneficial alleles within a single generation, including those that are of low frequency or absent in the population, while effectively bypassing linkage drag. For the dairy industry, genome editing can be used to make rapid genetic improvements that are precise, efficient, and transgene-free for functional traits that are not practically addressed without disrupting conventional breeding goals for overall economic merit based on genomic selection. Herein, various case studies for dairy cattle breeding are presented that demonstrate applications of genome editing for enhancing heat stress tolerance, reduced disease susceptibility, and other qualitative traits absent in some breeds. One case highlights the success of simultaneous editing of multiple loci through recent advancements in embryonic stem cell biology. Multiplexed editing is crucial for addressing the polygenic nature inherent to many economically important traits in livestock. However, maximizing the benefits of genome editing depends on the continued discovery of targets for editing that are commercially important. Commercialization also depends on rapidly evolving regulatory statutes for risk assessment, where some countries already permit the commercialization of cattle with non-GMO genome alterations through existing regulations. New breeding technologies such as genome editing are now poised to have significant impact in equipping elite performance cattle to be more resilient to infectious disease and climate change without the loss of production gains obtained from decades of selection.

## Highlights

• Genome editing enables the rapid and precise introgression of beneficial alleles.
• Bovine multiplexed editing has been successfully accomplished.
• Certain countries have allowed the commercialization of gene-edited cattle.

## Graphical Abstract

**Figure:**
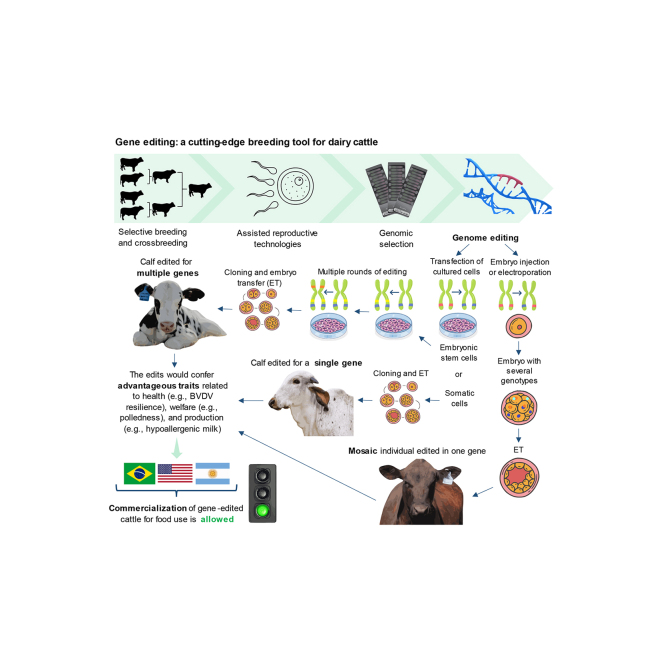
**Summary:** Conventional breeding methods have driven genetic progress in cattle. Genome editing, a novel breeding technique, can expedite the introduction of desirable traits into elite genetics in a single generation. Gene editing-mediated introgression of alleles has commonly been done with single-cell embryos, albeit often accompanied by the occurrence of mosaicism. In contrast, cloning using somatic cells effectively circumvents this problem but could come at the cost of lower pregnancy rates. The use of embryonic stem cells for cloning addresses both issues while allowing for multiplexed editing. Intended gene edits are usually target traits vital to the cattle industry, such as reduced disease susceptibility and heat tolerance. Remarkably, the United States, Brazil, and Argentina have allowed the commercialization of gene-edited cattle, with the latter 2 countries determining risk levels by the presence of transgenes. This differentiation based on risk-assessment criteria paves the way for the commercialization of genome-edited cattle and their associated products under conventional industry standards.

The global development of sustainable food systems has never been more pressing. The livestock industry plays an essential role in modern society by providing products such as meat and milk, which are rich in energy and premium-grade protein (Van Eenennaam, 2019). Conventional breeding programs remain the cornerstone of cattle genetic improvement, relying mainly on incremental genetic gains through selection for specific quantitative traits. Traditional breeding methods encompass selective breeding, crossbreeding, and assisted reproductive techniques such as cloning, embryo transfer, and artificial insemination. In recent years, advances in genome sequencing and genotyping have enabled the commercial use of genomic selection. An excellent example of this is the implementation of genomic selection in dairy cattle in the United States (Wiggans et al., 2017). The integration of these resources has resulted in improved genetic progress in animal breeding in developed economies.

Looking ahead at future challenges of selective breeding lies the imperative to develop livestock that are both more productive and better equipped to withstand the challenges posed by pathogens and climate change in local production environments. Recent global pandemics, such as avian flu and African swine fever, have underscored the far-reaching consequences of contagious livestock diseases on the disruption of animal protein markets. Traditionally, combating cattle diseases has relied on national and regional biosecurity and vaccination programs. Although these strategies have generally been successful in multiple geographies, they fall short of providing complete protection against some infectious agents, especially those lacking effective vaccines (Wang et al., 2022; Gao et al., 2023). Challenges such as these are further exacerbated by climate change, which intensifies heat-mediated stress, leading to increased disease susceptibility.

As such, livestock genetics needs to be at the forefront of solutions used to meet global food security challenges. Genome editing, also known as gene editing, is a technology based on the use of site-directed nucleases (**SDN**). These have been under research and development for the past 16 years as a breeding tool that introduces new traits within the cells of a food animal genome, through reprogramming of the DNA code. Some reprogramming can result in breeding outcomes that help producers better match their genetics to environmental conditions without sacrificing yield. For the sake of brevity and current commercial relevance, we excluded discussing any applications that involve transgenesis, as their adoption in the industry remains financially impractical due to significant challenges to reach commercialization even if the final product is a null segregant. To clarify, editing processes that involve transgenesis, also known as SDN3, involve the insertion of a DNA element containing a foreign gene with control elements (Friedrichs et al., 2019). Unlike genetically modified organisms (**GMO**) of the past, which typically involved the random incorporation of transgenes, genome editing offers unprecedented opportunities to introgress precise genome alterations (SDN1 and SDN2) that often are indistinguishable from those found naturally in an animal's genome. This capability to make single-generation genetic changes provides unique opportunities to address breeding for improved animal health and well-being.

Although conventional breeding methods, such as crossbreeding and selective breeding, can improve health and resilience to climate change and other environmental stressors, gains are often limited by difficulties in maintaining the best combinations of advantageous alleles for polygenic traits in future generations. Historically, selection for these types of traits has been limited by the cost and risk involved in phenotyping elite breeding animals for disease, lack of beneficial alleles in founding populations, and linkage with genetic variation antagonistic to overall genetic merit (Liu et al., 2022; Wang et al., 2022). Some of these factors explain why, to date, breeding goals for infectious and metabolic diseases have not been widely implemented through selection indices breeding programs (Bengtsson et al., 2022).

In response to these conventional breeding challenges, genome editing provides an alternative to introgressing known genetic alterations for improved health, adaptability, and resilience in a single generation. The concept of health encompasses the absence of disease and the preservation of the animals' overall physical and physiological well-being (Terranova and Laviola, 2004). Meanwhile, resilience refers to an animal's capacity to endure and recover from short-term perturbations caused by diseases and environmental stressors (Colditz and Hine, 2016). These elements directly affect the adaptability of cattle, a critical factor influencing their productivity and reproductive success (Wang et al., 2022).

For breeding of commercially viable animals, genome editing is a multifaceted process requiring quality management to standardize phenotypic outcomes. The main critical steps include the optimization of a genome editing tool for the specific target locus and the accurate and timely delivery of editing reagents to a cell type capable of producing a viable animal. The CRISPR-Cas9 technology stands out as one of the most transformative breeding tools (Horvath and Barrangou, 2010). This laboratory discovery was transformed from a bacterial defense system to be a tool providing specific DNA cuts using an RNA-guided Cas9 protein for any species with known genome sequence. The induced cuts prompt cellular enzymes to begin repairing the targeted genome through either non-homologous end-joining or homology-directed repair mechanisms. The former can cause small insertions or deletions, whereas the latter enables precise introgression specific to nucleotide-level changes (Vandana et al., 2021). One of the main cell types used for breeding a genome-edited animal is single-cell in vitro–fertilized bovine embryos. Here, editing reagents can be introduced by microinjection or electroporation. This approach has proven to be challenging for making exact nucleotide substitutions or obtaining complete homozygosity. Often, mosaic animals are produced, which require additional breeding to obtain non-mosaic offspring (Lin and Van Eenennaam, 2021). In contrast, somatic cell nuclear transfer (**SCNT**) allows for direct selection of cells with homozygous alterations at the on-target site. Unfortunately, outside of a few private service providers, SCNT is not generally a well-established method for animal production due to high pregnancy loss rates and adverse postpartum outcomes, likely due to incomplete epigenetic reprogramming of the cloned cell (Galli and Lazzari, 2021).

One recent alternative to improve cloning rates relies on the use of bovine embryonic stem cell (**bESC**) nuclear transfer. Recent advancements in bESC have made them feasible for genome editing (Bogliotti et al., 2018), with an enhanced capacity to undergo editing and nuclear transfer events compared with other cell sources used for cloning (T. S. Sonstegard and D. F. Carlson [Acceligen, Eagan, MN], unpublished data). It has been found that bESC have significantly improved longevity in culture and are similar to fibroblasts in SCNT blastocyst development rates, with reduced pregnancy loss and offspring abnormalities (T. S. Sonstegard and D. F. Carlson [Acceligen, Eagan, MN], unpublished data). Clearly, bovine genome editing relies on assisted reproductive technologies, and these recent advances using bESC are essential to the commercial deployment of health and well-being traits in cattle, especially those that rely on editing multiple loci.

After more than 10 years of experimental breeding, the first examples of gene-edited bovine traits for heat tolerance and reduced disease susceptibility have resulted in proven phenotypic outcomes with commercial value. Tropical environments have a major detrimental effect on most dairy breeds, particularly for milk yield and fertility due to heat stress. Heat stress compromises normal physiology, including immunity, which causes non-adapted cattle to be less productive and more susceptible to diseases (Bagath et al., 2019). The most promising gene editing target to mitigate heat stress in highly productive, temperate taurine cattle is prolactin receptor (*PRLR*), which when truncated causes *SLICK*. Six truncated variants (*SLICK* alleles) for *PRLR* have been identified in heat-tolerant Criollo breeds (Flórez Murillo et al., 2021). Animals harboring at least a single *SLICK* allele exhibit a distinct short and sleek hair coat phenotype referred to as the “slick phenotype.” The positive effects linked to the *SLICK*-associated phenotype have prompted efforts to introduce the *SLICK1* allele into dairy cattle in Puerto Rico, Florida, and New Zealand through crossbreeding (reviewed by Hansen, 2020). *SLICK* Holsteins graded up from Senepol × Holstein crosses had reduced body temperatures as well as lower respiration rates compared with traditional Holsteins, even when not in heat-stress conditions. Supporting evidence also indicates that *SLICK* mutations mitigate the detrimental effects of heat stress on milk yield and calving intervals. More recently, purebred Angus in Brazil (Bellini, 2018) and in the United States (Rodriguez-Villamil et al., 2021) were bred with *PRLR*-truncating mutations, through SCNT and embryo microinjection, respectively. Recently reported data demonstrate that gene-edited SLICK Angus significantly outperform non-edited contemporaries in regulating body temperature, body weight, scrotal circumference, and hair weight reduction (Cuellar et al., 2024). In all, *SLICK* mutations have been introgressed into 3 different breeds of beef and dairy cattle by gene editing, with regulatory decisions for commercial sales of semen, embryos, progeny, meat, and milk in US and Brazilian marketplaces.

Relative to edits that directly influence animal health, there was a recent breakthrough to improve disease resilience in cattle (Workman et al., 2023). The CD46 protein has been shown to be the principal host receptor governing bovine viral diarrhea virus (**BVDV**) infection (Maurer et al., 2004). In this case, Gir fibroblasts were treated with CRISPR/Cas9 reagents designed to alter 6 amino acids within the distal coding region of *CD46*. This genome alteration was shown to inhibit infection in both fetal cells and a lone juvenile animal. More importantly, no other changes causing unintended effects on animal physiology were detected, which is critical, as CD46 has multiple functions across many tissue types. Reduced BVDV susceptibility holds the potential to elevate animal welfare standards and conceivably reduce the need for antibiotics, as BVDV infections are known to increase the overall risk of secondary bacterial diseases in calves (Givens and Newcomer, 2015). This work is the first instance of a non-transgenic genome alteration in cattle that reduced the effects of a major viral disease. Earlier work had attempted to enhance bovine resistance to *Mannheimia haemolytica*-induced pasteurellosis, a disease causing significant economic losses in the cattle industry. Shanthalingam et al. (2016) successfully introduced a Q(−5)G substitution in both alleles of integrin subunit beta 2 (*ITGB2*) using ZFN, and the leukocytes from the edited fetus demonstrated the ability to inhibit *Mannheimia haemolytica* leukotoxin–induced cytotoxicity. However, editing of this strictly conserved amino acid among ruminants possibly had adverse effects on embryo development, as no live animals with this specific gene edit have been reported to date.

Repairing defective genes responsible for recessive lethal or heritable diseases is another application of gene editing based on a single-gene target model of breeding. This type of genetic improvement has the potential to eliminate inherited traits that compromise animal health and productivity. For example, the use of CRISPR/Cas9 in fetal (Ikeda et al., 2017) and skin (Ishino et al., 2018) fibroblasts to correct isoleucyl-tRNA synthetase (**IARS**) syndrome was reported in vitro. A recessive genetic disorder prevalent in Japanese Black cattle, IARS syndrome is attributed to the c.235G > C (p.Val79Leu) substitution in *IARS*, which impairs protein synthesis. Calves inheriting 2 copies of this SNP experience neonatal weakness, intrauterine growth delays, and an increased likelihood of perinatal mortality (Hirano et al., 2016). The extensive selective breeding for improved meat quality in Japanese Black cattle and widespread use of artificial insemination from a small pool of bulls have possibly increased the frequency of recessive deleterious mutations within the US population (Ikeda et al., 2017). Genome editing is an alternative to DNA marker-based selection that reduces variants causing genetic diseases in a single generation without detrimental effects on productivity and reduced biodiversity by breeding only non-carrier animals.

One last category of traits that is worth mentioning from those based on a single gene and that eliminate the need for management practices that can adversely affect animal well-being. These practices include either disbudding or dehorning to reduce injury risks to both cattle and farmers (Aldersey et al., 2020). One alternative is ramping up selection pressure on polled animals to increase the allele frequency in dairy breeds; however, the lower genetic merit of most existing polled sires, combined with inbreeding challenges and the commodity-based value of dairy semen, has hindered the widespread adoption of this approach (Mueller et al., 2019). Hence, genome editing to produce polled sires with high genetic merit is an innovative approach to improve dairy cattle welfare while maintaining productivity. To date, the first and best example of this in cattle was done through introduction of a causative variant of polledness in a proof-of-concept crossbred dairy animal (Carlson et al., 2016). In this case, the more than 1,000-year-old Pc mutation was introgressed via TALEN-mediated editing into a male fibroblast for cloning. As of late June 2024, polled dairy animals derived from genome editing treatment are still not on the market. However, there is promise that such dairy animals will be available in the future, following on from the first polled Wagyu cloned from “polled-edited” bESC, which were born in 2023 (T. S. Sonstegard, unpublished data). These animals prove it is possible to maintain the highest genomic merit for highly marbled beef while removing the horns in a single generation in a manner that could be considered practical and affordable for commercial purposes.

More recently, scientists in the field have been attempting to prove the feasibility of editing applications that could have a major influence on the commercial availability of elite genetics regardless of environment or management practices. This research has explored genome editing strategies to develop animals lacking germ-cell development genes, which generate host sires lacking their own reproductive cells. These surrogate males hold the potential to carry germ cells from exogenous high-merit donor bulls. This has been proven to be possible in male pigs and goats with nanos C2HC-type zinc finger 2 (*NANOS2*) knockout, in which donor-derived spermatogenesis was achieved (Ciccarelli et al., 2020). Proof of conception and adequate levels of sperm production were recently reported in *NANOS2* knockout cattle, suggesting advancement toward commercial development (Latham et al., 2023). A bull lacking germline cells has also been generated by disrupting nanos C2HC-type zinc finger 3 (*NANOS3*; Mueller et al., 2022). If the fertility rescue protocol for germline-ablated animals becomes scalable, this approach could be applied to create surrogate sires from breeds resilient to the pasture environment, potentially enabling wider distribution of superior non-adapted performance genetics through natural mating. Other applications of genetic engineering in the most elite dairy sires would be those to manipulate the male:female sex ratio of offspring (Van Eenennaam, 2019), which involve transgenesis or SDN3 and require a more thorough risk assessment by regulators globally. These applications are not included in this review, as they are beyond the scope of this manuscript.

All these previous instances illustrate the potential of genome editing to enhance cattle health or performance by editing a single target in the genome. The next-level power of genome editing to be leveraged on a commercial scale will require cell types such as bESC that can remain viable for cloning after editing multiple regions of the genome. Stacking traits is particularly relevant for addressing disease resilience, as these types of traits are usually underlaid by multiple alleles, necessitating several genomic alterations for a measurable phenotypic difference.

One such example is an ongoing project to breed adapted Holsteins for African smallholders. In this project, developing methods to introgress traits enhancing resilience to endemic tropical diseases and heat tolerance in Holsteins through multiplexed genome editing is a necessity to improve sustainability. The smallholder production environment needs environmentally durable animals combined with better milk yield potential, as these animals are the revenue generators and financial reserves for the household. In this project, the genome editing process was preceded by several stages, including high-value matings for in vitro fertilization embryo production, bESC development, pathogen testing, and genomic selection of “best bESC” for editing. This effort led to the generation of the world's first simultaneous triple and quadruple homology-directed repair-edited bovine clones (T. S. Sonstegard and D. F. Carlson [Acceligen, Eagan, MN], unpublished data). The genome-edited changes included the introgression of *SLICK* to improve heat tolerance and sequence variants found in 3 genes of native African cattle predicted to be associated with improved tolerance to disease caused by trypanosome and *Mycobacterium bovis* infections. These edits, based on naturally occurring alleles from breeds under natural selection, are eligible to be considered non-GMO for regulatory frameworks in Nigeria and Kenya.

The commercial use of genome editing tools varies between economies based on the regulatory laws and statutes in place to evaluate the risk of either the genome editing process or the product, or both. Many countries have already established or are drafting regulations that distinguish transgene-free, gene-edited animals from GMO. Countries with regulations that classify the commercialization of gene-edited animals without transgenes as non-GMO include Argentina, Brazil, Japan, Australia (indels only by process), Nigeria, Ethiopia, Colombia, Paraguay, India, and Kenya (Ledesma and Van Eenennaam, 2024).

In the United States, the Food and Drug Administration's (**FDA**) determination of low-risk enforcement discretion for 2 edited Angus introgressed with the *SLICK* trait (Rodriguez-Villamil et al., 2021) marked a milestone for intentional genomic alterations in food-producing animals (US FDA, 2022). This determination represents a regulatory stance in which the FDA, although recognizing that certain gene-edited animals could fall under its oversight, chooses not to enforce the full spectrum of regulatory requirements based on current understanding of the risks involved. This revision to the previous regulatory guidance at FDA is now officially approved by the US Office of Management and Budget (US FDA, 2024). The SLICK Angus animals mentioned above with FDA enforcement discretion (CTNBio, 2022), and more recently a Holstein bull (CTNBio, 2023) with edited *SLICK* alleles (T. S. Sonstegard, unpublished data), received CTNBio classifications as non-GMO in Brazil.

These gene-edited bovine animals, developed by Acceligen Inc., and their offspring can now enter the commercial marketplace and food supply chains from the United States and Brazil under the standards applied to animals bred through conventional methods. Likewise, their genetic material can be marketed, which is also the case for the semen from a Nelore bull with disrupted myostatin (*MSTN*) for increased meat yield (Proudfoot et al., 2015); this semen was considered non-GMO in Brazil (CTNBio, 2021). Based on these cases, it is predicted that the number of countries distinguishing between GMO (transgenic) and gene-edited animals, thereby allowing the more rapid commercialization of the latter, will continue to grow in the coming years.

In the past decade, the messaging about the importance of global food security has continued to intensify, with a growing population and climate change driving the need for sustainable animal production to meet rising protein demands. The benefits offered by biotechnologies to improve animal well-being and health should lead to the social license of genome editing as an important breeding tool to sustain livestock production of protein (McConnachie et al., 2019). To increase the potential benefits of genome editing, research should also continue identifying genetic variants affecting traits of economic importance. Genome editing has often involved disrupting single genes, but the true potential lies in precise genetic alterations for variants with known phenotypic outcomes, which can be maximized through multiplexed editing.

Selective breeding, enhanced by genomic selection and in combination with advanced reproductive technologies, has greatly accelerated the rate of genetic improvement in cattle production. This toolset has yielded, and will continue to yield, substantial benefits for animals, farmers, and consumers. The addition of genome editing to the animal breeder's toolbox could accelerate genetic gain, drawing on genetic variation from other populations, mimicking mutations from other species, and incorporating rationally designed beneficial alleles for better health and well-being. This latter approach, possibly non-GMO in some markets, has enabled the introduction of reduced susceptibility to BVDV into cattle, which was previously impossible with traditional breeding. Remarkably, the editing of multiple genes underlying health and resilience traits has also been achieved, with a resultant healthy set of Holstein animals attesting to its feasibility. Commercialization of gene-edited animals still entails navigating through the complexities of regulatory approvals in different economies, despite multiple examples of low-risk assessments of gene-edited animals outside the United States.
